# Cyanobacteria as Nanogold Factories: Chemical and Anti-Myocardial Infarction Properties of Gold Nanoparticles Synthesized by *Lyngbya majuscula*

**DOI:** 10.3390/md16060217

**Published:** 2018-06-20

**Authors:** Esam M. Bakir, Nancy S. Younis, Maged E. Mohamed, Nermin A. El Semary

**Affiliations:** 1 Chemistry Department, College of Science, King Faisal University, Al-Ahsa 31982, Saudi Arabia; 2 Chemistry Department, College of Science, Ain Shams University, Cairo 11566, Egypt; 3 Pharmaceutical Sciences Department, College of Clinical Pharmacy, King Faisal University, Al-Ahsa 31982, Saudi Arabia; Nyounis@kfu.edu.sa (N.S.Y.); Memohamed@kfu.edu.sa (M.E.M.); 4 Pharmacology Department, Zagazig University, Zagazig 44519, Egypt; 5 Pharmacognosy Department, College of Pharmacy, Zagazig University, Zagazig 44519, Egypt; 6 Biological Sciences Department, College of Science, King Faisal University, Al-Ahsa 31982, Saudi Arabia; Nelsemary@kfu.edu.sa; 7 Botany and Microbiology Department, Faculty of Science, Helwan University, Cairo 11795, Egypt

**Keywords:** cardiac injury, cardiac biomarkers, cyanobacteria, gold nanoparticles, myocardial infarction

## Abstract

To the best of our knowledge, cyanobacterial strains from the Arabian Gulf have never been investigated with respect to their potential for nanoparticle production. *Lyngbya majuscula* was isolated from the AlOqair area, Al-Ahsa Government, Eastern Province, Kingdom of Saudi Arabia. The cyanobacterium was initially incubated with 1500 mg/mL of HAuCl_4_ for two days. The blue-green strain turned purple, which indicated the intracellular formation of gold nanoparticles. Prolonged incubation for over two months triggered the extracellular production of nanogold particles. UV-visible spectroscopy measurements indicated the presence of a resonance plasmon band at ~535 nm, whereas electron microscopy scanning indicated the presence of gold nanoparticles with an average diameter of 41.7 ± 0.2 nm. The antioxidant and anti-myocardial infarction activities of the cyanobacterial extract, the gold nanoparticle solution, and a combination of both were investigated in animal models. Isoproterenol (100 mg/kg, SC (sub cutaneous)) was injected into experimental rats for three days to induce a state of myocardial infarction; then the animals were given cyanobacterial extract (200 mg/kg/day, IP (intra peritoneal)), gold nanoparticles (200 mg/kg/day, IP), ora mixture of both for 14 days. Cardiac biomarkers, electrocardiogram (ECG), blood pressure, and antioxidant enzymes were determined as indicators of myocardial infarction. The results showed that isoproterenol elevates ST and QT segments and increases heart rate and serum activities of creatine phosphokinase (CPK), creatine kinase-myocardial bound (CP-MB), and cardiac troponin T (cTnT). It also reduces heart tissue content of glutathione peroxidase (GRx) and superoxide dismutase (SOD), and the arterial pressure indices of systolic arterial pressure (SAP), diastolic arterial pressure (DAP), and mean arterial pressure (MAP). Gold nanoparticles alone or in combination with cyanobacterial extract produced an inhibitory effect on isoproterenol-induced changes in serum cardiac injury markers, ECG, arterial pressure indices, and antioxidant capabilities of the heart.

## 1. Introduction

Cyanobacteria are oxygenic prokaryotic phototrophs which are widespread in almost all types of habitats worldwide [1]. Cyanobacteria present in the eastern region of the Kingdom of Saudi Arabia are diverse in their niches; some are marine, whilst others are freshwater types [2]. The Arabian Gulf is reported to host several cyanobacterial genera, most commonly those belonging to filamentous forms.

Over the last three decades, a number of microorganisms and higher plants have been found to be competent, eco-friendly nano-factories for the synthesis of nanoparticles through mechanisms adapted by the organism to cleanse their environment from heavy metals and radionuclides [3]. Amongst biological systems used, cyanobacteria and microalgae attract special attention since they not only have the ability to convert many toxic metals into non-hazardous physical and chemical forms including nanoparticles, but also have some other advantages over other organisms. Cyanobacteria and microalgae grow rapidly in rather inexpensive growth media and produce a large amount of biomass in a short time by converting CO_2_ into organic matter and releasing oxygen during photosynthesis, thereby cleaning up the environment and at the same time bioremediating toxic metals. Some cyanobacteria and algal genera such as *Plectonema boryanum* and *Sargassum wightii* have been reported for their ability to bioconvert effect of Au^3+^ to Au^0^ and the subsequent formation of gold nanoparticles (GNPs) [4,5]. Nanoparticles can be formed either by extracellular or intracellular enzymes [6]. The reduction of gold ions may follow a similar pattern to silver ions, which possibly proceed through reductase enzymes and electron transporters as quinones, where the electrons produced are used to avoid metal ion damage in the presence of enzymes such as NADH-dependent reductases D [7]. However, the intracellular formation of nanoparticles causes an imbalance in nutrient and substance exchange processes [8]. The biological route for nanoparticles synthesis helps to avoid other chemical and physical routes with hazardous and toxic processing conditions and by products as well as allowing expensive synthesis of nanoparticles at physiological pH, temperature, and pressure. Moreover, nano-products synthesized through a biological route are expected to be biocompatible, therefore minimizing environmental and human health risks.

Metal nanoparticles are involved in many medical applications and possess a variety of pharmaceutical and pharmacological properties. One of the most outstanding applications for metal nanoparticles is their use in labeling and imaging, especially in magnetic resonance imaging, surface-enhanced Raman scattering, and fluorescence spectroscopy [9]. Another popular use of metal nanoparticles is as optical or electrochemical biosensors [10,11]. The use of metal nanoparticles in such techniques enabled their involvement in the medical and clinical diagnosis of many diseases such as cancer [12], Alzheimer’s disease [13], HIV [14], hepatitis B [15], tuberculosis [16], diabetes [17], and influenza [18]. Metal nanoparticles have also been used as a treatment for many disorders and medical conditions such as cancer [19,20,21] and rheumatoid arthritis [22,23], and for several topical and systemic infections as anti-microbial agents [24,25,26]. Metal nanoparticles are now recognized as an excellent drug delivery system due to their high biocompatibility and excellent conjugation ability with biological material such as DNA and RNA. Nanoparticles show good optical properties, enabling drug tracking and bioavailability studies [9,27,28,29,30]. Working as drug vectors, metal nanoparticles can exhibit drug-targeting or gene-delivering properties [9].

Myocardial infarction, a consequence of ischemic heart disease, is a leading cause of mortality worldwide. Many medicinal agents are now used to manage this condition depending on varied mechanisms of action including dissolving thrombosis and repair of infarcted area myocytes. The treatment of ischemic heart diseases, including infarction using metal nanoparticles, is a new approach and is little studied. Ahmed et al. [31] have investigated the effect of metal nanoparticles on ischemia induced in the heart of experimental rats and the results indicated the curative activity of the nanoparticle on the infarcted heart.

To the best of the authors’ knowledge, there is no data available about the use of cyanobacteria from the eastern region of Saudi Arabia for the production of nanoparticles, despite their wide biodiversity. The main aim of this manuscript was to investigate the ability of the cyanobacterium *Lyngbya majuscula* to produce GNPs from externally supplied bulk ionic gold salt solution. The anti-myocardial infarction activity of the produced GNPs was investigated using a novel approach in which different treatments were prepared, including the cyanobacterial extract, GNP solution, and a combination of both.

## 2. Materials and Methods

### 2.1. Isolation and Characterization of the Cyanobacterium Lyngbya majuscula from the Arabian Gulf Region

**Figure 1:**
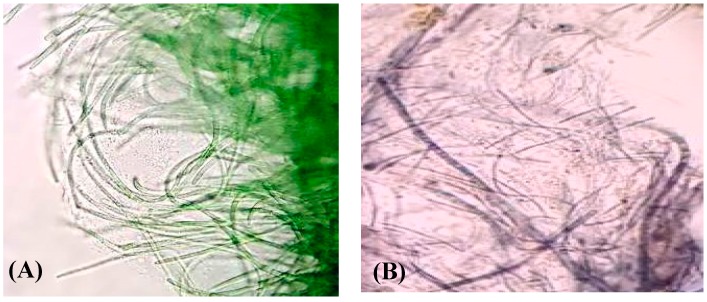
Optical images of (**A**) green cyanobacterium *Lyngbya majuscula* and (**B**) the same cyanobacterium in the mode of gold nanoparticle production.

Water samples were collected from AlOqair beach, Al-Ahsa Governorate, Eastern Province, Kingdom of Saudi Arabia. Isolation of monocyanobacterial cultures was performed using F/2 medium [32]. The characterization of the cyanobacterial identity as a filamentous strain was performed microscopically. The filamentous, non-heterocystous cyanobacterium was identified as marine *Lyngbya majuscula* (Figure 1).

### 2.2. Synthesis of Gold Nanoparticles by the Isolated Lyngbya majuscula

Cyanobacteria-mediated biogenic synthesis of gold nanoparticles was checked by visual observation of the experimental set with time. Thus, cyanobacterial culture (5 mL) was treated with 1500 mg/mL Au^3+^ (HAuCl_4_) solution immediately after the subculture process. The nanoparticle suspensions produced were scanned using UV-visible spectroscopy (540 nm). The efficiency of production of GNPs (as a percentage) was calculated as 13.3% through recording the percentage of yield of the reduction process. The concentrations of HAuCl_4_ and GNPs were 1500 mg/mL and 200 mg/mL, respectively, after 24 h according to Lenartowicz et al. [33] using the following equation:

The percentage of yield = GNP concentration (mg/mL)/gold salt concentration (mg/mL) × 100

### 2.3. Characterization of Gold Nanoparticles Produced

#### 2.3.1. Scanning Electron Microscopy (SEM)

The surface characteristics of gold nanoparticles from cyanobacteria samples were studied by scanning electron microscopy (SEM) on a Phillips CM201C microscope (Phillips, Eindhoven, The Netherlands) at 80 kV connected with an energy dispersive X-ray spectrometer (EDX).

#### 2.3.2. FTIR Analysis

FTIR spectra of cyanobacteria and gold nanoparticles complexes with cyanobacteria were measured by a Bruker Alpha FT-IR spectrophotometer.

#### 2.3.3. Cyclic Voltammetry

Electrochemical analyses were performed using a NuvantEZstat Pro potentiostat in a three-electrode system with a standard Ag/AgCl reference electrode (prepared in saturated KCl solution), Pt-wire counter electrode, and gold working electrode (size diameter, 2 mm). The cyanobacteria and cyanobacteria–GNP complex samples were measured at the scan rate 100 mV·S^−1^ without pH adjustment or supporting electrolytes due to the salt-containing nature of the samples [34,35].

### 2.4. Determination of the Anti-Myocardial Infarction Activity

#### 2.4.1. Materials

Isoproterenol (ISO) (Cat. No I6504), and creatine phosphokinase ELISA kits (Cat. No. C3755-3.5KU) were obtained from Sigma-Aldrich (St. Louis, MI, USA). Cardiac tropinine T ELISA kits (Cat. No. mbs162871) were purchased from My BioSource, Santiago, CA, USA, while creatine kinase—myocardial bound ELISA (Cat. No. ABIN955837), glutathione peroxidase assay (ab102530) and superoxide dismutase activity assay (ab65354) kits were purchased from Abcam Co., Ltd. (Hong Kong, China). Additional chemicals and reagents used were of the uppermost analytical rank acquired from commercial sources.

#### 2.4.2. Extraction of the Cyanobacteria *Lyngbya majuscula*

The cyanobacteria were filtered from a 60-day-old culture using Whatman No 1 filter paper, and the filtered cells were allowed to dry in a desiccator (Silica Gel) until constant weight was obtained. The dried biomass was extracted using methanol 100% (100 mL per 1 gm dry bacteria). The organic solvent was filtered, then the filtrate was centrifuged for 5 min (4000× *g*). The supernatant was dried under reduced pressure and the residue was dissolved in DMSO to give 200 mg/mL stock solution. 

#### 2.4.3. Preparation of Gold Nanoparticle Solution for Animal Application

The GNPs were freshly filtered from the cyanobacteria, and then the gold concentration was determined using the diameter and absorbance [36]. The concentration of GNP solution was adjusted to give a stock solution of 200 mg/mL

#### 2.4.4. Animals

Forty-eight male Sprague–Dawley rats (170 ± 25 g) were purchased from the animal house facility, King Saud University, Riyadh, and kept in standard laboratory conditions (23 °C ± 1 °C), maintained on a standard commercial rodent diet using a 12 h light/dark cycle during the accommodation period. All animal experimental procedures and protocols were approved by the Animal Research Ethics Committee at King Faisal University, and they were performed in accordance with the Guidelines for the Ethical Conduct for Use of Animals in Research, King Faisal University.

#### 2.4.5. Experimental Design

Rats were allocated into eight groups (six rats per group, *n* = 6) after acclimating to the facility. Groups 1–4 were given SC (saline injection), while groups 5–8 were injected with isoproterenol (100 mg/kg, SC). The injections were given once a day for three days in both sets. Following the three-day injection period, Group 1 was treated as a control group (Normal) and injected with normal saline (IP) for 14 successive days. Groups 2 and 3 were given cyanobacterial extract (200 mg/kg/day, IP) and GNPs (200 mg/kg/day, IP), respectively, for 14 successive days (Control BE and Control GNP groups). Group 4 was given a mixture of cyanobacterial extract and GNPs (200 mg/kg/day each, IP) for 14 successive days (Control BE/GNPs group). Group 5 was treated as an isoproterenol control group (ISO control) and was injected with normal saline (IP) for 14 successive days. Groups 6 and 7 were given cyanobacterial extract (200 mg/kg/day, IP) and GNPs (200 mg/kg/day, IP), respectively, for 14 successive days (BE and GNPs groups, respectively). Group 8 was given a mixture of cyanobacterial extract and GNPs (200 mg/kg/day, each, IP) for 14 successive days (BE + GNPs group).

#### 2.4.6. Electrocardiogram (ECG) and Blood Pressure (BP) Recording and Measurement

After 14 successive days, urethane-anesthetized rats (1.5 g/kg) were placed in a prone position on a board and an ECG was continuously recorded using noninvasive computerized ECG apparatus from Kent Scientific (Torrington, CT, USA). Five minutes later, the ECG was recorded for five seconds. Heart rate, R-R and QT intervals, R wave amplitude, and ST segments were calculated from ECG recordings by computer. BP measurements were done, using a noninvasive computerized tail-cuff system from Emka Technologies’ systems (Paris, France) which consists of placing a cuff on the animal’s tail to occlude the blood flow. The pressure was raised and then slowly released. The cuff pressure when the pulse signal reappeared was taken as the systolic pressure. And the pressure when the pulse signal level recovered its initial value was recorded as diastolic pressure.

#### 2.4.7. Tissue Handling and Biochemical Estimation

After 14 successive days, blood samples were collected and centrifuged for 10 min at 4000 rpm to separate serum, which was then stored at −80 °C prior to analysis. Anesthetized animals were then sacrificed by cervical dislocation. For biochemical parameters, subsamples of heart tissue were homogenized, with phosphate buffer saline (50 mmol L^−1^, pH 7.4). The acquired homogenates (10% *w/v*) of different investigational groups were centrifuged (12,000 rpm/20 min/4 °C), and the collected supernatants were used for determination of different biochemical parameters. 

#### 2.4.8. Determination of Cardiac Marker Enzymes

ELISA kits, following the manufacturer’s instructions, were used to measure serum levels of creatine phosphokinase, cardiac tropinine T, and creatine kinase—myocardial bound.

#### 2.4.9. Estimation of Antioxidant Activity

Heart tissue supernatants were used to evaluate the endogenous antioxidative enzymes activities, comprising glutathione peroxidase (GSHPx) and superoxide dismutase (SOD) using standard assay kits and a microplate reader (VERSAmax™, Molecular devices, Sunnyvale, CA, USA).

### 2.5. Statistical Analysis

All the values were expressed as mean ± SEM (*n* = 6). Image analysis was completed using Image J software. Graph Pad Prism 5 software was performed to evaluate the statistical analysis. The value *p* < 0.05 was considered statistically significant using one-way analysis of variance (ANOVA) followed by Tukey’s test.

## 3. Results

### 3.1. Synthesis of Gold Nanoparticles by the Isolated Lyngbya majuscula

**Figure 2:**
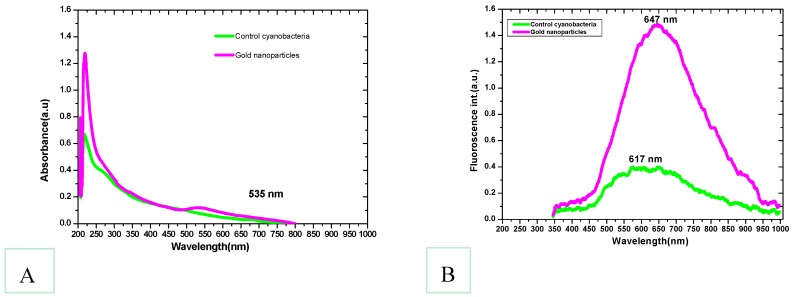
(**A**) The UV-visible absorption (**B**) and the fluorescence spectra of **control cyanobacteria** (*Lyngbya majuscula* cyanobacteria culture with no Au^3+^ addition, green line) and produced **gold nanoparticles** (*Lyngbya majuscula* cyanobacteria culture supplied with Au^3+^, purple color). The absorption band seen at 535 nm indicates the production of gold nanoparticles.

Within 24 h, the cyanobacterial biomass treated at 1500 mg/mL Au^3+^ solution began to develop light purple coloration in several places. The appearance of the purple color signified the bioconversion of Au^3+^ to Au^0^ and formation of GNP nanoparticles. As time proceeded, all the experimental biomass turned dark purple (Figure 1). In contrast, the control set biomass remained blue-green as before. The purple color was developed due to the surface plasmon resonance of the resultant gold nanoparticles as a band appeared at about 535 nm (Figure 2) indicating the production of gold nanoparticles. A gradual increase in purple coloration in the cells indicated that the steady synthesis of GNPs is most likely to be a function of time. In addition, the color change of the *L. majuscula* growth medium to purple indicated extracellular synthesis of gold nanoparticles. From earlier studies, it is evident that under similar conditions, the formation of purple color of the biomass would be due to the reduction of Au^3+^ to Au^0^ and subsequent formation of gold nanoparticles at intra and extracellular levels [4]. When the bacterial culture suspension was scanned by UV-visible spectroscopy, a band appeared at about 535 nm (Figure 2) which indicated the gold surface plasmon resonance (SPR) and thus the production of gold nanoparticles. The SPR peak corresponds to aggregation of the gold nanoparticles in the solution, which were possibly produced by biomolecules, proteins, and enzymes on the surface of cyanobacterial cells.

**Figure 3:**
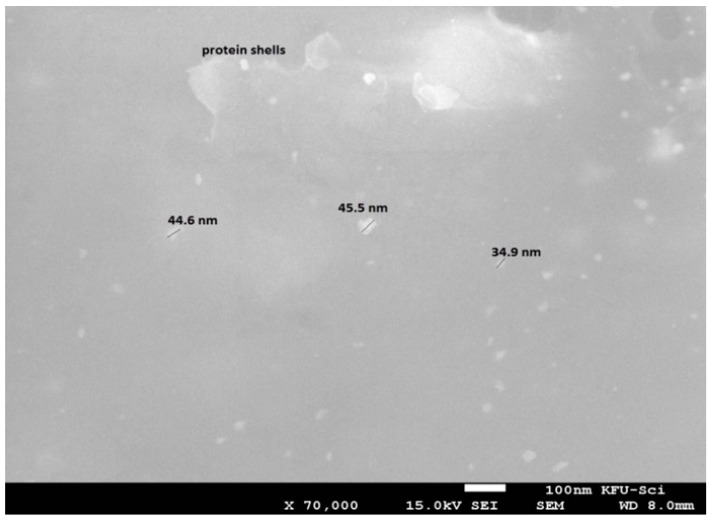
Scanning electron microscope image of gold nanoparticles produced by cyanobacteria *Lyngbya majuscula* with average size diameter 41.7 ± 0.2 nm.

In general, the surface plasmon band of gold nanoparticles is visible between 510 and 560 nm in aqueous suspension and it fluctuates with varying morphologies of gold nanoparticles [37]. Absorption bands beyond 600 nm can also emerge in addition to the shorter wavelength oscillation due to the presence of non-spherical nanoparticles. The higher PL (photoluminescence) emissions of gold nanoparticles around 650 nm are due to the surface plasmonic effect—collective oscillations of the 6sp band free electrons as explained in Mie theory [38]. Scanning electron microscope (SEM) images of the present study confirmed the presence of discrete nanoparticles in the sample as shown in Figure 3. *Lyngbya majuscula* was observed to produce spherical nanoparticles of average size diameter 41.7 ± 0.2 nm. SEM images confirmed the presence of nanogold particles and were used for determination of their size, which served the purpose of the study and confirmed the ability of cyanobacteria to biosynthesize gold nanoparticles. The presence of protein shells around some nanoparticles was suggested (Figure 3); however, future studies can be conducted using TEM to confirm protein shell presence. The occurrence of some large and extremely small nanoparticles supports the intrinsic polydispersity generally obtained in the case of biogenic synthesis [39].

### 3.2. Energy-Dispersive X-ray Spectroscopy (EDX Images)

**Figure 4:**
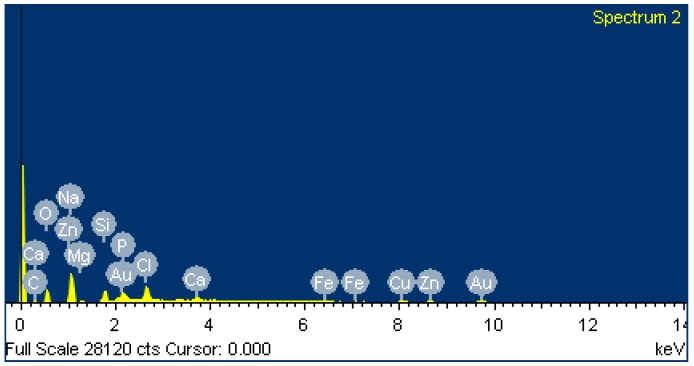
The energy-dispersive X-ray spectroscopy (EDX) spectrum of gold nanoparticles produced by the cyanobacteria *L. majuscula*.

Cyanobacteria are rich in minerals as they form an integral part of the cell. In the present study, higher concentrations of magnesium were observed in all of the species as it is a key component of chlorophyll molecules. Heavy metals such as copper, iron, manganese, nickel, mercury, cadmium, zinc, lead, and molybdenum are essential micronutrients required for the growth of cyanobacteria, and in higher concentrations they may have an inhibitory effect on growth [34,35]. EDX analysis determined the percentage of GNPs as ~9% in *L. majuscula* composition (Figure 4).

### 3.3. FTIR Spectra

**Figure 5:**
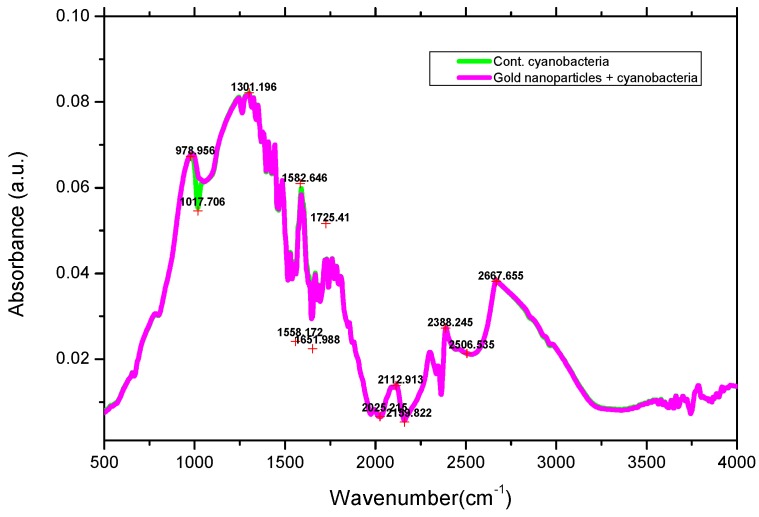
FTIR analysis of gold nanoparticle suspension complex with *L. majuscula*.

FTIR spectra of the protein of *L. majuscula* conjugated complex with gold nanoparticles showed the characteristic amide II and I bands at 1665 and 1582 cm^−1^ (Figure 5). These amide absorption bands did not show shifts from their non-conjugated spectra, suggesting that the amide moieties (C=O and N–H) do not take part in production of the GNP-conjugated complexes. This also suggests that any inter or intra-molecular H-bonding that these functionalities may be involved in is not affected by complexation with the GNPs. Furthermore, no significant band shape or wavenumber shift is observed in the –OH absorptions of these compounds either before or after GNP complex formation, suggesting that the –OH moieties do not play a role in conjugate complex formation and H-bonding does not appear to be a part of the complex formation. Shifts and changes in the C=O absorption band also suggest possible H-bonding changes with complex formation [40]. The absorption bands at ~1022 cm^−1^ are due to C–N stretching vibrations of amines [41]. These results indicate the presence of protein shells around the GNPs, which was observed in SEM studies as well.

### 3.4. Cyclic-Voltammetry

**Figure 6:**
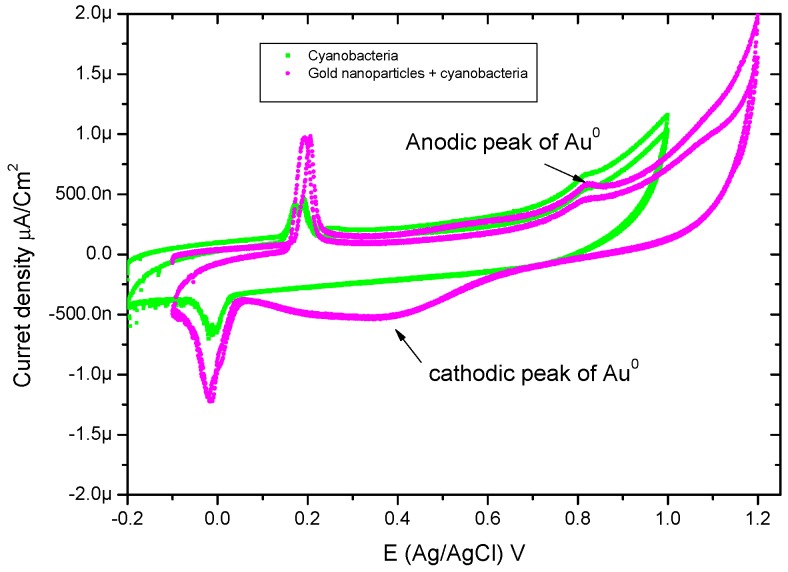
Acyclic voltammetry scan was recorded at the rate of 100 mV/s for cyanobacteria and gold nanoparticle complexes with cyanobacteria.

The cyclic voltammetry scans, recorded at the rate of 100 mV/s, for cyanobacteria and GNPs complexes with cyanobacteria, respectively, are shown in Figure 6. Cyanobacteria have anodic and cathodic peaks around 0.2 V and −0.014 V (vs. Ag/AgCl), respectively. The oxidation peak of gold nanoparticles appeared at 0.8 V, and this is attributed to the rapid re-oxidation of GNPs due to the absence of exogenous capping agent [42]. The mechanism of extracellular reduction of Au^3+^ to Au^0^ nanoparticles occurred by the pilus on the surface (endogenous glycogen) of a microbe is capable of carrying out the role of electron transfer as a type of electric wire [43]. One mechanism of metal nanoparticles biosynthesis by microorganisms is bio-reduction. In microbial bio-reduction processes, myriads of proteins, carbohydrates, and bio-membranes are involved [44]. Nanoparticles are formed on cell wall surfaces, and the first step in bio-reduction is the trapping of the metal ions on this surface. This probably occurs due to the electrostatic interaction between the metal ions and positively charged groups in enzymes present at the cell wall. This may be followed by enzymatic reduction of the metal ions, leading to their aggregation and the formation of nanoparticles [38]. The microbial cell reduces metal ions by use of specific reducing enzymes like NADH-dependent reductase or nitrate-dependent reductase [45].

### 3.5. Effect of Cyanobacterial Extract, GNPs and their Combination on Cardiac Marker Enzymes

**Figure 7:**
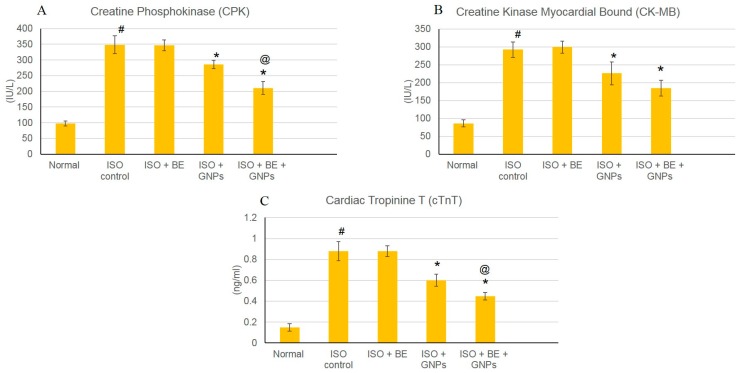
The treatment effects of bacterial extract (BE, 200 mg/kg/day, IP) and gold nanoparticles (GNPs, 200 mg/kg/day, IP) and combination (BE + GNPs) respectively, for 14 successive days on isoproterenol (ISO)-induced myocardial infarction with respect toserum level of cardiac marker enzymes. (**A**) CPK, (**B**) CP-MB, and (**C**) cTnT in normal and isoproterenol-induced MI rats. All values were expressed as mean ± SD (*n* = 6).ISO: isoproterenol, BE: bacteria extract, GNPs: gold nanoparticles, CPK: creatine phosphokinase, CK-MB: creatine kinase-myocardial bound, andcTnT: cardiac troponin T. # indicatesa statistically significant difference from the normal group, * indicatesa statistically significant difference from the isoproterenol control group, @ indicatesa statistically significant difference from the gold nanoparticles-treated (*p* < 0.05) group using one-way ANOVA followed by Tukey’s test as a post hoc analysis.

The effects of isoproterenol (ISO) and cyanobacterial extract (200 mg/kg/day, IP) and GNPs (200 mg/kg/day, IP), respectively, for 14 successive days (BE and GNPs) and their combination atthe mentioned doses on numerous cardiac indicator enzymes (creatine phosphokinase (CPK), creatine kinase—myocardial bound (CP-MB), andcardiac troponin (T (cTnT)) in normal and myocardial infarcted (induced via isoproterenol) tissues are illustrated in Figure 7. The generation of myocardial infarction subsequent to isoproterenol administration significantly augmented cardiac indicator enzymes (CPK, CK-MB, and cTnT) (Figure 7A–C) as compared with normal rats. Treatment with GNPs (200 mg/kg/day, IP) alone, and the combination of BE/GNPs with the mentioned doses, significantly ameliorated the isoproterenol-induced escalation of the diagnostic cardiac indicator enzymes (CPK, CK-MB, and cTnT), in comparison with isoproterenol-control rats. However, there were no significant alterations detected in the cardiac indicator enzymes in rats in the Control BE, Control GNP, and Control BE+GNP groups, nor in those in treatment with bacterial extract (200 mg/kg/day, IP) alone.

### 3.6. Effect of Cyanobacterial Extract, GNPs and Their Combination on Heart Rate and Blood Pressure Indices Recording and Measurement

**Figure 8:**
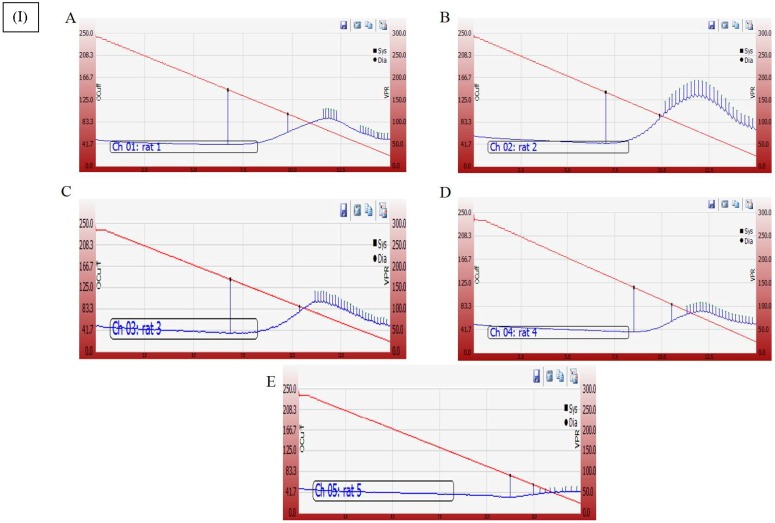

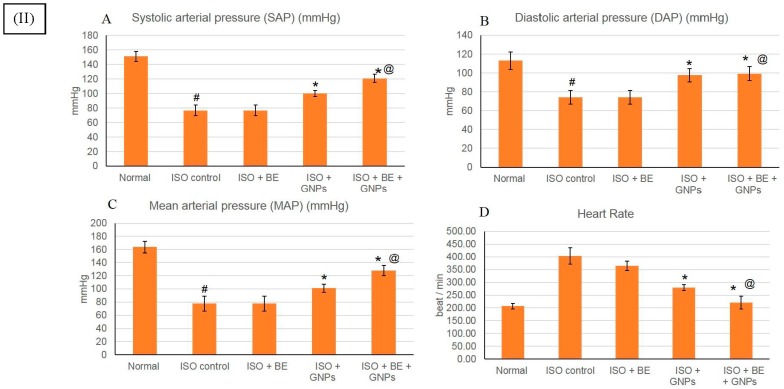
(**I**) Tracings obtained during blood pressure (BP) recordings. (**A**): Normal, (**B**): ISO control, (**C**): ISO + BE, (**D**): ISO + GNPs, (**E**): ISO + BE + GNPs. (**II**) The treatment effects of bacterial extract (BE, 200 mg/kg/day, IP) and GNPs (200 mg/kg/day, IP) and their combination (BE + GNPs), respectively, for 14 successive days on isoproterenol-induced myocardial infarction (MI) with respect to SAP(**A**), DAP (**B**), MAP (**C**), and heart rate(**D**)in normal and ISO-induced MI rats. All values were expressed as mean ± SD (*n* = 8).ISO: isoproterenol, SAP: systolic arterial pressure, DAP: diastolic arterial pressure, MAP: mean arterial pressure, and HR: heart rate. # indicates a statistically significant difference from the normal group, * indicates a statistically significant difference from the isoproterenol control group, @ indicatesa statistically significant difference from the gold nanoparticle-treated (*p* < 0.05) group using one-way ANOVA followed by Tukey’s test as a post hoc analysis.

ISO injections induced a significant escalation in the heart rate and decline in systolic arterial pressure (SAP), diastolic arterial pressure (DAP), and mean arterial pressure (MAP) as compared to the normal control group (Figure 8). However, treatment with GNPs (200 mg/kg/day, IP), and the co-administration (BE, and GNPs) with the mentioned doses significantly (*p* < 0.05) prevented the ISO-induced decline of the arterial pressure indices SAP, DAP, and MAP. Similarly, the increase in heart rate (HR) was also attenuated in GNP and BE+GNP rats as compared to the ISO control rats.

### 3.7. Effect of Cyanobacterial Extract, GNPs and Their Combination on Electrocardiographic Trace Recording and Measurement

**Figure 9:**
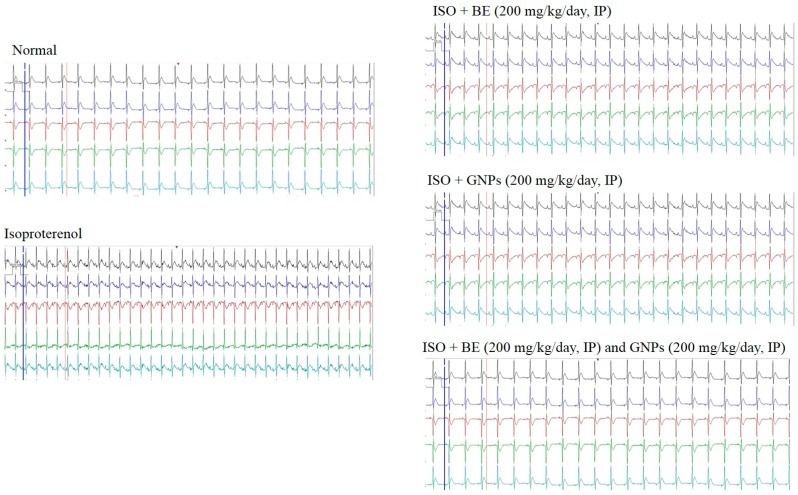
Electrocardiogram (ECG) tracings that were recorded for evaluating heart rate and rhythm disorders. Bacterial extract (BE, 200 mg/kg/day, IP), gold nanoparticles (GNPs, 200 mg/kg/day, IP), combination (BE+GNPs), isoproterenol (ISO).

A representative electrocardiographic trace of normal and experimental animals is shown in Figure 8. Normal control, Control BE, Control GNP and Control BE/GNP-treated rats showed normal ECG patterns, whereas rats treated with isoproterenol showed a significant increase in the ST segment and QT intervals. Conversely, a significant decrease in QRS complex, and P-R and R-R intervals was seen on comparison with control rats, indicative of infarcted myocardium (Table 1). Treatment with GNPs (200 mg/kg/day, IP) and (BE, and GNPs), respectively, for 14 successive days showed a reverse in ECG-induced alterations (Table 1 and Figure 9).

### 3.8. Effect of Cyanobacterial Extract, GNPs and Their Combination on Lipid Peroxidation and the Activities of Antioxidant Enzymes in ISO-Induced MI in Rats

**Figure 10:**
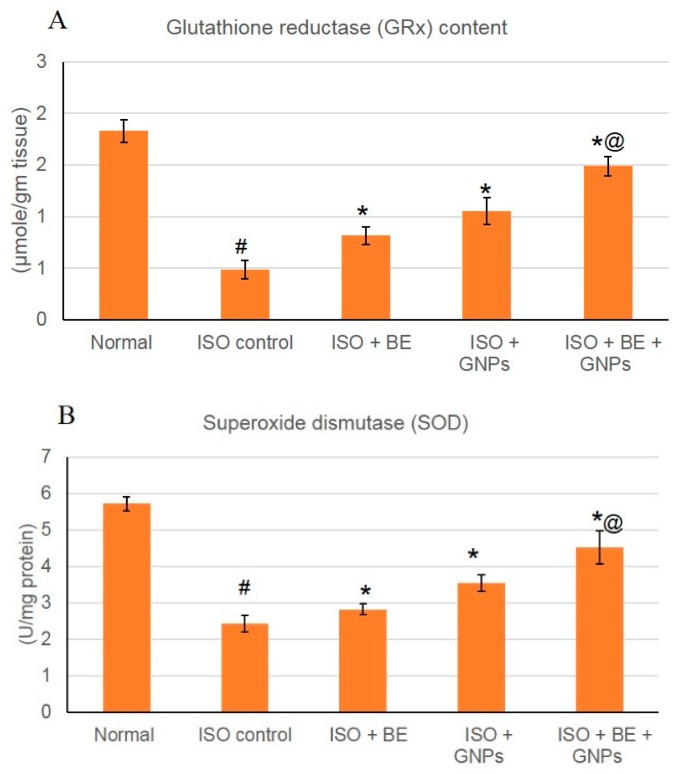
The treatment effects of bacterial extract (BE, 200 mg/kg/day, IP) and GNPs (GNPs, 200 mg/kg/day, IP) and their combination for 14 successive days in isoproterenol (ISO)-induced myocardial infarction (MI) on GRx (**A**) and SOD (**B**) in normal and ISO-induced MI rats. All values were expressed as mean ± SD (*n* = 8).ISO: isoproterenol, GRx: glutathione reductase, and SOD: superoxide dismutase. # indicates a statistically significant difference from the normal group, * indicates a statistically significant difference from the isoproterenol control group, @ indicates a statistically significant difference from the gold nanoparticle-treated group (*p* < 0.05) using one-way ANOVA followed by Tukey’s test as a post hoc analysis.

The activities of the antioxidant enzymes GRx and SOD were decreased (*p* < 0.05) significantly in the rats injected with ISO when compared to the control. However, treatment with bacterial extract (200 mg/kg/day, IP), GNPs (200 mg/kg/day, IP), and BE and GNPs in combination, respectively, for 14 successive days in rats with isoproterenol-induced myocardial infarction resulted in a significant increase in the activity of antioxidant enzymes GRx, and SOD when compared to ISO-treatment alone (*p* < 0.05), Figure 10. In rats from the Control BE, Control GNPs, and Control BE+GNPs groups, no significant differences in the activities of the antioxidant enzymes were detected when compared to the control.

## 4. Discussion

The Arabian Gulf is a relatively small marine water body. Its salinity level is on the increase (nearly greater than 40 ppt) [46]. This is due to its nature as a semi-closed water body as well as the fact that several huge desalination plants are operating in its coastal region. They desalinate water and dispose of the highly concentrated brine back into the Arabian Gulf, thereby increasing the salinity level greatly. The condition is exacerbated by the high water evaporation rate. This means that there is a great input of ions that is added to that water body. Another overlooked factor is ballast water that is being disposed of into the Arabian Gulf. Oil tankers from different parts of the world upload oil and dispose of ballast water containing contaminating residues into the Arabian Gulf. Only organisms with high ability to cope with such a contaminated and salinized habitat are able to survive. Other environmental challenges faced by those organisms are high solar irradiance and the nearly stagnant state of the water. Given all of these factors, it seems that cyanobacteria growing in such a niche have a rather unique metabolic activity that has enabled them to grow and detoxify harmful ions through reducing them into non-harmful nanoforms [47,48].

With regard to the ability of the cyanobacterium *L. majuscula* to reduce gold ions to metallic nanogold particles both intra- and extracellularly, Chakraborty et al. [49] reported this phenomenon, which starts with metabolic-independent binding, followed by accumulation, and then reduction. Parial and Pal [50] indicated the initial formation of gold nanoparticles intracellularly, followed by subsequent formation extracellularly. The reports assumed that abiotic factors, for example, presence of reducing moieties such as reducing sugars in the polysaccharide sheath and fatty acids in the plasma membrane, or other cellular reducing entities, might be involved in reducing gold ions. Biotic factors such as the involvement of reducing enzymes in reduction of gold cannot be excluded [47].

As for extracellular-reducing activities, it is reported that parts of the polysaccharide sheath can dissociate from the filaments, forming what is known as exopolysaccharide into solution. This exopolysaccharide is known for its heavy metal-removing activity [51] and its high content of reducing sugars that can be effective in reducing gold nanoparticles.

The surface plasmon resonance of gold nanoparticles is a result of interaction between oscillating electric fields of a light ray with the free electrons causing a concerted oscillation of electron charge that is in resonance with the frequency of visible light. As particle size increases, the wavelength of surface plasmon resonance-related absorption shifts to longer wavelengths. Red light is then absorbed, and blue light is reflected, yielding solutions with a pale blue or purple color.

The surface-active molecules on the cell surface of the cyanobacterium are suggested to be involved in the reduction of metal ions. However, their number may be not high enough to allow for the reduction of all ions at higher concentrations, i.e., the number of bioactive molecules and the number of cells present may be limiting factors for the number of ions to be reduced. Therefore, the synthesis of nanoparticles may be dependent on metal concentration as well as the number of the cells or the number of bioactive molecules present. This differential response indicates the possibility of custom designed nanoparticles by varying cell number and metal concentration in solution.

Environmental applications of nanogold related to color changes associated with their aggregation and/or local refractive index change have been exploited as optical sensing methods for the detection of toxins [52], heavy metals, and other environmental pollutants [53] in water, soil, and other environmental samples. GNPs are also utilized to enhance the performance of electrochemical sensors due to their catalytic properties [54]. Electrochemical sensors have been widely investigated for environmental pollutants screens.

There are certain factors that can affect the efficiency and impact of nanoparticles, which include their size, shape, and distribution. Those factors are affected by synthesis procedures, reducing agents, and stabilizers. For example, small-sized nano-silver particles are more efficient as antimicrobial agents, probably due to ease of penetration of cellular membranes. The recently reviewed antibacterial modes of action of silver nanoparticles were found to be related to the induction of alterations of many cellular functions such as membrane permeability, respiratory activity, and DNA replication [55]. With regard to GNPs, their antibacterial activity against acne or scurf is reported and they have commercial applications in soap and cosmetic industries. They can remove waste materials from skin and control sebum. GNP-mediated growth inhibition of different Gram-positive and Gram-negative bacteria and fungi has been recently reported [56]. Functionalizing GNPs with polyethylene glycol increases stability, both in vivo and in vitro [57]. The inert, non-toxic GNPs can be bio-synthesized in a range of sizes from 1 to 150 nm and can be readily functionalized with targeting ligands and drugs to allow delivery at the required site by way of their photophysical properties or by intercellular glutathione levels [57]. Many hypotheses discussing the mechanism of nanoparticles formation by cyanobacteria [58] suggested the involvement of the water-soluble pigment phycocyanin in the reduction of silver ions and/or other organic molecules, most likely polysaccharides. All were able to produce nanoparticles, reinforcing the hypotheses that both extracellular and intracellular moieties are able to reduce silver ions. Another hypothesis that cannot be excluded is the involvement of reducing enzymes such as NADH dependent reductases.

*Lyngya majuscula* is a non-heterocystous filamentous cyanobacterium that is found in tropical and subtropical waterbodies. It is a well-known source of bioactive compounds producing an array of biologically active metabolites, which can be increased by factors such as stage of growth and light. The cyanobacterial extract is rich with a variety of these compounds, and a number of reviews have described some of these compounds and their activities [59,60]. Examples of those compounds are thiazole peptides (pseudodysidenin, nordysidenin), barbamide (mixed polypeptide–polyketide), pseudodysidenin, nordysidenin, and apramides (linear peptides), and lyngbyapeptin A [61,62,63].

The induction of myocardial infarction in a rat model using isoproterenol presents a non-invasive methodology for investigation of potential cardioprotective agents. Isoproterenol is a synthetic catecholamine, which induces myocardial infarction in rat models through several mechanisms such as increased water content, amplified oxidative stress, and infiltration of inflammatory cells to damaged areas [64]. In the study herein, isoproterenol succeeded in the induction of myocardial infarction condition in rats as shown by elevated cardiac marker enzymes, irregularities in the heart rate, blood pressure and ECG parameters, and the depletion of the anti-oxidant enzymes (GRx and SOD). Treatment with cyanobacterial extract alone had no significant effect on the elevated cardiac marker enzymes, as can be seen from Figure 7, Figure 8 and Figure 9 and Table 1, however; it showed a noteworthy enhancement in antioxidant activity, as can be seen from Figure 10. *L. majuscula* possesses high diversity of active metabolites belonging to several classes of phytochemicals, and the cyanobacterium retains many pharmacological properties such as neurotoxic, cytotoxic, antimicrobial, and antiprotozoal activities [65]. However, no metabolic miscellany and pharmacological actions were sufficient to induce the treatment of the myocardial infarction disorder. GNPs have been produced by *L. majuscula* as a result of environmental incorporation of the gold ions by the cyanobacterium. The use of such GNP solutions alone or in combination with the cyano- bacterium has achieved effective management for cardiac infarction, as can be seen from the amendment of cardiac marker enzymes, reversal of ECG irregularities caused by isoproterenol, normalization of arterial pressure indices, and the elevation in antioxidant enzymes.

Although GNPs alone showed anti-myocardial infarction activity, addition of the cyanobacterial extract intensified this effect. Bearing in mind that the cyanobacterial extract did not show any activity on the ischemic heart, this synergetic activity could be explained through the presence of certain phytochemicals in the cyanobacterial extract that protect GNPs or alter their pharmacokinetics in the body including metabolism and excretion. For in vitro stabilization of GNPs, different agents are utilized, including thiols, surfactants, citrate, or phosphorus-containing ligands [66]. As mentioned above, *L. majuscule* comprises a high diversity of phytochemicals, including bioactive peptides [59,61,62,63] (in particular thiazole peptides). Sulfur-containing compounds may act as a shield for GNPs surfaces through weak physical or strong chemical bonds [67]. Therefore, it might be assumed that thiazole peptides in the bacterial extract may have some protective effect on the GNPs produced. Furthermore, the study strongly suggested the presence of a protein shell around the produced GNPs. Part of this protein shell could be attributed to the proteins and peptides found in the cyanobacteria itself. Continued supply of such proteins and peptides (in the form of cyanobacterial extract) could help the protection of the produced GNPs. In addition, the presence of such a shell around the GNPs can affect their metabolism in the body as well as their excretion rates, resulting in a prolonged and better effect. However, all the above is speculation based on some facts related to the cyanobacterium and GNPs, and further investigation is required. 

The effect of gold nanoparticles on myocardial infarction was not intensively investigated previously [31], and the effect of GNPs against doxorubicin-induced heart failure has been proven [68]. However, the current study may add to the pool of knowledge on the activity of GNPs in myocardial infarction and tissue repair. To the best of the authors’ knowledge, this is the first study to discuss the myocardial infarction activity of GNPs produced by cyanobacteria and *L. majuscula* in particular.

## Figures and Tables

**Table 1:** The treatment effects of bacterial extract (200 mg/kg/day, IP), GNPs (200 mg/kg/day, IP), and a combination (BE and GNPs) respectively, for 14 successive days on isoproterenol-induced myocardial infarction with respect to SAP (A), DAP (B), MAP (C), and HR (D) in normal and ISO-induced MI rats. All values are expressed as mean ± SD (*n* = 8).ISO: isoproterenol, SAP: systolic arterial pressure, DAP: diastolic arterial pressure, MAP: mean arterial pressure, and HR: heart rate. # indicates a statistically significant difference from the normal group, * indicates a statistically significant difference from the isoproterenol control group.

	Normal	ISO Control	ISO + BE	ISO + GNPs	ISO + BE+ GMPs
ST elevation (mV)	0.027 ± 0.002	0.184 ± 0.013 #	0.145 ± 0.029	0.064 ± 0.007 *	0.060 ± 0.009 *
QRS complex (s)	0.042 ± 0.001	0.027 ± 0.002 #	0.027 ± 0.002	0.038 ± 0.001 *	0.042 ± 0.000 *
QT interval (s)	0.045 ± 0.004	0.088 ± 0.008 #	0.069 ± 0.005	0.061 ± 0.003 *	0.061 ± 0.004 *
P-R interval (s)	0.232 ± 0.023	0.160 ± 0.003 #	0.197 ± 0.028	0.212 ± 0.019 *	0.222 ± 0.016 *
R-R interval (s)	0.230 ± 0.031	0.151 ± 0.004 #	0.180 ± 0.003	0.199 ± 0.010 *	0.208 ± 0.014 *
